# Editorial Etiquette

**Published:** 1850-11

**Authors:** 


### ARTICLE V.

### EDITORIAL ETIQUETTE.

We owe the *Charleston Medical Journal* an apology for the omission of credit for an article in our last issue. Probably from neglect, quite a number of such omissions occur in our exchanges of late, in reference to original matter taken from our pages. Most of these are placed in their extract departments showing that they are not original, and we will not specify them. But we observe in the New York Medical Gazette our editorial notice of the Concour held in Rush Medical College for Demonstrator of Anatomy, without any thing to show that it was not making its first appearance there. And in the Ohio *Medical and Surgical Journal* our article on the use of Collodion in Erysipelas, by Dr. Freer, looked so much like an original one that the New Jersey Medical Reporter copied and credited it to that journal. This is the second complaint of the kind we have made against the last named journal within a year.

